# Supplementary material for: Are carbon emissions trading and green financial instruments synergistic? -Comprehensive quantitative research based on content analysis
Source: PLoS One. 2024 Mar 7;19(3):e0298601. doi: 10.1371/journal.pone.0298601 (PMC10919601; doi:10.1371/journal.pone.0298601)
Supplement: S1 Data — (ZIP) [file pone.0298601.s001.zip › Data/地级市Z_F工作报告.zip!/║╙─╧/╚2├┼╧┐╩╨/└┤╫╘╣1⁄2╓┌║┼í╛┬φ┐╦╩2╛▌═°í┐_2010.docx]

各位代表：

现在，我代表市人民政府向大会作政府工作报告，请予审议，并请各位政协委员提出意见。

一、2009年工作回顾

刚刚过去的2009年，是新世纪以来我市经济社会发展面临困难最大、挑战最为严峻的一年，也是全市上下积极应对国际金融危机取得重大成效的一年。市政府在省委、省政府和市委的正确领导下，在市人大、市政协的监督支持下，坚持以党的十七大和十七届三中、四中全会精神为指导，深入贯彻落实科学发展观，团结带领全市人民，全力以赴战危机，千方百计促“三保”，在逆境中实现了经济社会平稳较快发展，圆满完成了市五届人大四次会议确定的各项任务。

（一）经济平稳较快增长。预计全年地区生产总值完成706亿元，比上年增长12%。地方财政一般预算收入完成41.5亿元，增长13.1%；一般预算支出完成85.6亿元，增长30.9%，“十件实事”等民生支出得到有效保障。预计规模以上工业增加值完成377亿元，增长13%。城镇居民人均可支配收入13510元，增长9%；农民人均纯收入5010元，增长7%。节能减排成效明显，万元生产总值能耗预计下降5%；化学需氧量、二氧化硫排放量分别控制在省定目标1.8万吨和14万吨以内。

（二）项目建设势头强劲。全社会固定资产投资预计完成545亿元，其中城镇固定资产投资预计完成470亿元，增长36.5%。强力实施项目建设年活动，全市“8155”投资促进计划确定的138个超亿元重点项目，已建成投产59个，在建71个，完成投资274.8亿元，项目数量、投资规模、建成数、开工率均创近年来之最。列入省考核重点项目、省“1074”工业结构调整项目、省“8511”投资促进计划项目分别提前3个月、2个月、1个月完成省下达投资目标。产业集聚区建设加快推进，7个省级产业集聚区已建成面积37.34平方公里，入园企业1029个，从业人员16.4万人。

（三）工业经济企稳回升。企业服务年活动深入开展，帮助企业协调解决难题724个，争取省电解铝储备计划7万吨，启动氧化铝闲置产能120万吨、电解铝闲置产能28万吨。规模以上工业增加值自去年5月份以来连续保持两位数增速，停产半停产企业由年初的186家下降到41家，占全部658家规模以上企业的6.2%。引导企业实现产销对接，省、市企业对接会签约资金44.06亿元。加大融资力度，开通全省首家中小企业金融服务网，成立担保机构28家，形成55亿元担保能力；金融机构贷款余额264.3亿元，较年初实际新增60.84亿元，新增额是上年的2.8倍，增长额及增速均创历史同期最高水平。

（四）产业结构逐步优化。农业基础地位继续巩固，粮食生产再获丰收，总产62.8万吨。特色农业不断壮大。灵宝苹果入选“河南省最具影响力十大地理标志产品”，全市果品总产162万吨，浓缩果汁出口5.5万吨，比上年增加1.8万吨；完成烟叶收购68.2万担，成为全省烟叶第一大市；林业产值大幅增长，冬季造林面积占全省的1/5。林果、畜牧、烟叶等特色产业占农业总产值的比重达85%。工业支撑地位明显增强。五大支柱产业占全市规模以上工业产值的比重达75%；工业内部升级加快，56个重大产业升级项目扎实推进，恒生科技、思睿科技等一批自主创新能力强、市场前景好的高新技术企业不断壮大。服务业发展迅速。文化旅游持续升温，函谷关通过国家4A级景区验收，全市接待国内外游客978万人次，实现国内旅游收入54.84亿元；消费市场繁荣活跃，社会消费品零售总额预计完成171亿元，增长19.1%；商贸物流产业迅猛发展，大中海商业文化广场等项目开工建设，家乐福超市等一批国际、国内知名大型连锁企业将落户我市；房地产市场日趋繁荣，建业、天盛等知名房地产企业投资我市，中原世纪苑等房地产项目进展顺利。

（五）开放合作成效显著。积极开展大招商活动，先后参加和举办了香港、上海、杭州、厦门、深圳、宁波、台湾和第15届国际黄河旅游节暨投洽会等一系列招商活动，共签订协议169个，签约总金额达1003.6亿元。利用外资工作取得新突破，引进省外资金项目93个，实际到位资金80.2亿元，超额完成省定目标；实际利用外资预计可达2.6亿美元，绝对值居全省第3位。大通关体系建设步伐加快，中国三门峡海关申报工作进展顺利，国家铝及铝制品质量监督检验中心开工建设，中国河南出入境检验检疫局三门峡办事处综合楼主体封顶。金融环境进一步优化，政银企合作不断加强，在全省率先与工农中建四大银行签订战略合作协议，到位资金192.59亿元，超额完成年度计划。豫晋陕黄河金三角区域协调发展综合试验区纳入国家中部崛起规划；与中国贸促会、中国黄金集团、省地矿局、省有色地矿局、省移动公司、省电力公司等建立了稳定的战略合作关系。

（六）城乡发展更加和谐。“四城联创”扎实推进，创建国家园林城市成果得到验收专家组的肯定。环境综合整治取得新成效，市区空气质量优良天数达到327天。新区规划启动，郑西高速铁路新三门峡站及站前广场、大岭桥等一系列市政重点项目竣工，市文体中心、中心医院病房楼等工程稳步推进。加快“三纵四横”大交通网络建设，郑西高速铁路试运行，运三铁路项目建议书通过国家发展改革委批复，黄河公铁两用桥建设在即，连霍高速改扩建工程进入征地拆迁阶段。城中村改造进展较快，累计完成投资3.8亿元。新农村建设稳步推进，全市1/3村庄规划通过专家评审，开工建设新型农村住宅社区37个；完成农田水利基本建设投资4.66亿元，22座病险水库除险加固工程进展顺利，新增和改善灌溉面积10万亩。

（七）社会事业全面进步。省、市“十件实事”圆满完成。群众关注的儿童福利院、阳光小学、城市集中供热二期、迎宾大道、廉租住房等建成投用，扶贫搬迁完成859户、3727人，解决了16.96万人的饮水安全问题，建成县乡公路499.7公里、通村公路244.3公里，新增户用沼气2.6万户。城市信用社成功改制，三门峡市商业银行和湖滨农村商业银行正式挂牌营业。社会保障能力增强，全民创业氛围浓厚，养老失业保险金统筹额、城镇新增就业人数、下岗失业人员再就业等超额完成年度任务，被确定为全国工伤预防试点城市和全省工伤保险市级统筹试点城市。科技创新步伐加快，科技对经济发展的贡献率达到52.7%；全市高新技术产业总产值完成23.25亿元，增长15.07%。教育事业健康发展，职业教育园区规划启动，外国语高中开工建设，普通高招成绩明显。成功举办市六运会，87个行政村建成“农民体育健身工程”，市体育局荣获全国群众体育先进单位。文化事业持续发展，成功创建中国楹联文化城市，市文化局获得全国文化系统先进集体，原创现代曲剧《大山的女儿》、改编传统蒲剧《孙安动本》荣获全省金奖。全面完成第三次全国文物普查田野调查工作。医疗卫生条件不断改善，完成12所乡（镇）卫生院改建任务。新农合参合率达97.2%，甲型H1N1流感得到有效控制。人口计生工作继续加强，人口自然增长率控制在5‰以内。城乡社会救助力度加大。加强应急管理体系建设，各类突发事件得到妥善处置。食品药品安全不断加强，公众饮食和用药安全得到保障。严格落实安全生产责任制，深入开展安全生产“三项行动”和“安全生产年”活动，加强重点领域和行业的安全监管，安全生产形势趋于稳定。扎实开展“信访积案化解年”活动，坚持信访突出问题周会审、领导包案和群众工作日制度，全市信访形势总体平稳。加强社会治安综合治理，平安三门峡建设跃上新台阶，再次荣获全国综治优秀市，成为全省唯一蝉联这一荣誉的省辖市，2009年度又获省平安建设、信访工作先进省辖市。

（八）精神文明和民主法制建设稳步推进。精神文明创建工作力度加大，社会文明程度不断提升。坚持依法行政，自觉接受市人大及其常委会的依法监督，支持政协参政议政，办理人大代表意见建议74件、政协委员提案248件，满意或基本满意率达到100%。政府自身建设不断加强，市、县政府机构改革稳步推进。扩大基层民主，第三届社区居委会换届选举工作圆满完成。继续深入开展民主评议机关作风效能活动，加强惩治和预防腐败体系建设，廉政建设和反腐败斗争取得新成果。国防教育和双拥工作进一步加强，优抚安置成效突出，民兵预备役工作有了新提高，驻市解放军和武警部队在处置突发事件、维护社会稳定、支持地方建设等方面作出了突出贡献，实现省级双拥模范城“六连冠”。审计、物价、侨务、对台、档案、史志、人防、气象、无线电、民族宗教、妇女儿童、河务移民、地震应急、残疾人、老龄等工作都取得了新成绩。援助江油恢复重建工作进展顺利。

过去一年的成绩，是全市在遭受国际金融危机严重冲击、面对重重困难与挑战的特殊背景下取得的，的确来之不易。在这场应对危机的重大考验中，我们既取得了一定成绩，也积累了在复杂经济环境中推动经济社会发展的实践经验。认真回顾总结，主要有以下四点体会：一是必须坚持科学发展为先。以深入学习实践科学发展观活动为载体，坚持把应对危机的成果作为衡量学习实践活动成效的试金石，紧紧抓住金融危机中暴露出来的资源依赖性过高、产业链条短、结构不合理等不符合科学发展观要求的矛盾和问题，深挖思想根源，果断采取措施，勇闯发展新路，在战危机、调结构的实践中提高贯彻落实科学发展观的自觉性和坚定性，有力推动了经济发展方式的转变，保持了经济平稳较快发展的良好态势。二是必须坚持改善民生为本。将积极应对危机与全力改善民生有机地结合起来，切实把扩大民生投入作为战危机、扩内需、促消费的重要抓手，集中大量的人力、物力、财力，扎实办好省、市“十件实事”等惠及民生的大事、要事，妥善解决涉及群众切身利益的热点、难点问题，保证了群众生活质量在危机冲击面前不下降、有提升。三是必须坚持谋划长远为要。面对金融危机，我们见事早，行动快，科学判断，主动出击，准确把握危机影响我市的特点，迅速启动以项目建设为主体、招商引资和政银企合作为支撑的“一体两翼”经济刺激计划，扎实开展项目建设年和企业服务年活动，引导企业抱团取暖，并通过多种方式动员全市人民提振信心，共克时艰，为夺取应对危机的最终胜利抢占了先机、赢得了主动；科学谋划结构调整和经济转型，为长远发展奠定了基础。四是必须坚持狠抓落实为基。把狠抓各项措施的落实作为应对危机的基础性工作，以“两转两提”为切入点，以健全机制为着力点，以落实责任为关键点，大力弘扬焦裕禄精神和求真务实、艰苦奋斗作风，分块把守，重点突破，凝心聚力，协调联动，加大政府执行力，增强工作实效性，为圆满完成全年目标任务提供了坚强保障。

这些来之不易的成绩，是省委、省政府和市委正确领导的结果，是全市上下坚定信心、危中求机，齐心协力、扎实苦干的结果，饱含着全市人民的心血与汗水，凝聚着社会各界的辛苦和智慧。值此，我代表市人民政府，向全市广大工人、农民、知识分子、干部、人民解放军指战员、武警官兵、公安民警以及社会各界人士致以崇高的敬意和衷心的感谢！

同时，我们也清醒地看到，政府工作与人民的期望还有一定差距，前进道路上还存在一些不容忽视的困难和问题。一是当前经济持续回升的基础还不牢固，影响发展的不确定因素依然存在；二是发展方式粗放、经济结构不尽合理、产业层次不高、竞争力不强的深层次矛盾还没有得到根本解决，调整产业结构、促进经济转型的任务还很艰巨；三是工业经济增长压力较大，资金紧张矛盾比较突出；四是城乡公益事业和基础设施欠账较多，建设任务非常繁重；五是政府职能和作风转变还不到位，党风廉政建设需要进一步加强。对此，我们要采取针对性措施，认真加以解决。

二、2010年政府工作总体要求和奋斗目标

今年是实施“十一五”规划的最后一年。做好今年政府工作，对夺取应对危机全面胜利、保持经济平稳较快发展、为“十二五”规划启动实施奠定良好基础具有十分重要的意义。当前，虽然我们还没有完全走出金融危机的阴影，但是全市经济企稳回升势头强劲，广大干部群众干事创业的热情高涨，科学发展的体制机制正在形成，我们已经度过了最艰难的时期，只要全市上下团结一心，锐意进取，就一定能够夺取今年经济社会发展的新胜利！

今年政府工作的总体要求是：全面贯彻党的十七大和十七届三中、四中全会精神，以邓小平理论和“三个代表”重要思想为指导，深入贯彻落实科学发展观，以“调整产业结构，加快经济发展方式转变，实现经济持续平稳较快发展”为主线，以经济结构战略性调整为重点，加快推进工业化、城镇化、农业现代化，着力深化项目带动、强化企业服务，着力深化改革开放、加强科技创新，着力保障改善民生、促进社会和谐稳定，抢抓机遇，奋力拼搏，全面完成“十一五”规划目标任务，努力开创经济社会跨越式发展新局面。

今年经济社会发展的主要目标是：全市生产总值增长11%。地方财政一般预算收入增长11%。全社会固定资产投资增长21%。社会消费品零售总额增长15%。外贸出口增长11%；实际利用外资增长15%。居民消费价格涨幅3%左右。城镇居民人均可支配收入增长8%；农民人均纯收入增长7%。人口自然增长率控制在6.5‰以内。城镇登记失业率控制在4.5%以内。单位生产总值能耗降低5%，化学需氧量和二氧化硫排放量分别控制在1.78万吨和13.5万吨以内；发展方式转变和经济结构调整取得重大进展。

三、加快经济结构战略性调整，提高经济发展的质量和效益

（一）坚持“一条主线”。坚持“调整产业结构，加快经济发展方式转变，实现经济持续平稳较快发展”这条主线，注重“调结构”，在强化工业支撑、做强特色农业的基础上，合理优化产业布局，加快产业结构调整，大力发展现代服务业，调优一产、调强二产、调大三产，实现三次产业全面协调发展；注重“促转型”，大力发展绿色经济、循环经济、低碳经济，加快发展集约集群经济，加强节能减排和生态环境建设，努力建设资源节约型和环境友好型社会；注重“增效益”，加快科技成果转化，拉长产业链条，提高产品附加值，发挥产业集聚效应，增强经济的内生动力和活力，提高经济发展的质量和效益。

（二）深化“两大活动”。即深化项目建设和企业服务两大活动。巩固项目建设年活动成果，围绕省十大产业振兴规划等一系列重大规划，结合“十二五”规划编制，高起点谋划一批事关经济社会发展大局的高精尖项目。大力发展循环经济型、高新技术型、精深加工型、先进制造型、劳动密集型产业，在产业升级、高新技术、自主创新、文化旅游、现代农业、节能减排等关键领域，建设超亿元项目160个以上，投资总规模1000亿元以上，当年完成投资300亿元以上。做大做强支柱产业。优化能源结构，提高资源利用效率，开工建设三门峡火电厂三期、华能2×30万千瓦热电联产等项目，着力打造全省重要的能源基地。加强有色金属的精深加工，开工建设义煤集团30万吨高精度铝板带箔等项目，不断拉长产业链条。加快重化工基地建设，推进义煤1000万方煤制气、盛源化工30万吨醋酸等项目，不断提高煤化工产品附加值。加快推进林果加工业，抓好湖滨果汁6万吨功能饮料扩建等项目，促进农业增效、农民增收。不断壮大新兴优势产业。利用我市能源、原材料优势，加快装备制造业、汽车零部件产业发展，加快推进河南金马新型电频调速节能电机车等项目，开工建设三门峡迦南地科技100万套发动机节能环保装置等项目，使其成为调整产业结构的生力军。大力培育战略先导产业。以发展新能源、新材料、新技术为重点，新上一批生物、化工、医药、电子信息等高新技术产业项目，重点抓好鹏飞电子节能灯、地久矿业7000吨多晶硅一期等项目，打造高新技术产业链和特色产业群，培育新的经济增长点。加快发展现代服务业。突出抓好现代物流、文化、旅游、金融等产业发展，促进现代制造业与服务业的有机融合、互动发展。高度重视文化旅游业项目建设，开工建设虢国文化产业园、仰韶文化产业园，推进函谷关古文化旅游区开发等续扩建项目。加快现代商贸物流业项目建设，促进房地产业健康发展，抓好大中海商业文化广场等项目，努力打造豫晋陕黄河金三角重要商贸物流中心。

继续坚持企业服务年活动的好经验、好做法，全力以赴抓服务、解难题、促转型、保态势。加强经济运行调节。健全经济运行预警机制，加强对煤、电、油、运、资金等主要生产要素和重点产业、重点企业的监测分析。建立经济运行协调机制，引导煤电铝等上下游企业合作联动。加强市场监管，保持物价基本平稳。加强扭亏增盈工作，不断提高企业效益。破解企业发展难题。全力实施省“企业服务行动计划”，继续完善落实骨干企业领导分包制度，及时帮助解决存在困难和问题。认真落实优惠政策，积极向上争取支持，全力帮助停产半停产企业恢复产能，千方百计提高企业开工率。积极开展产销对接活动，鼓励企业扩大出口规模，大力开拓国内外市场。推动节约集约发展。强化资金保障，扩大直接融资规模，大力吸引民间投资，加强对企业、项目的资金支持，缓解企业融资难问题。强化国土资源保障，坚持最严格的耕地保护制度和节约用地制度，大力推进土地综合整治，确保重点建设用地。实施节能攻坚计划，推进全民节能减排，推广节能技术和设备，加快淘汰落后产能，坚决取缔浪费资源、污染环境的“十五小”、“新五小”等列入国家淘汰目录的企业。强化环境保护和生态建设，继续加强重点流域、重点区域、重点行业污染综合整治和饮用水水源地保护，推广清洁生产示范项目建设，大力发展节能环保产业。加快自主创新体系建设。认真实施省“自主创新体系建设工程”，加快构建企业为主体、市场为导向、产学研相结合的技术创新体系。加快自主创新平台建设，建成灵宝黄金股份科技研发中心。大力实施重大科技专项，突破一批事关经济社会发展全局的核心技术。实施质量兴市战略，鼓励企业加大研发力度，促使柠檬酸金钾等具有自主知识产权的项目加快进度、扩大规模，培育一批高新技术、创新型企业。注重人才在自主创新中的关键作用，培养、引进技术创新骨干和青年创新人才。推进科技体制机制创新，拓宽科技成果转化渠道。提升企业综合竞争力。突出产业转型升级、企业战略重组和培育壮大市场主体等重点，加快实施转型升级重点企业发展规划和项目。支持龙头骨干企业做大做强，鼓励中小企业主动参与上下游产业的纵向合作和横向联合，增强抵御市场风险的能力。

（三）强力推进“四区建设”。一是科学发展示范区（城市新区）建设。围绕建设优美宜居城市，科学调整城市总体规划，抓紧启动新区建设，以郑西高速铁路新三门峡站启用为带动，规划旅游休闲、高档住宅、现代商务、研发基地、高新技术产业等功能区，提高城市集约节约发展水平。以城乡统筹为抓手，创新管理体制，完善政策措施，引导产业和人口集聚，实现以产带城、以城促产，使新区成为城市建设的新亮点、产业发展的新高地和经济发展新的增长极。二是产业集聚区建设。按照“企业集中布局、产业集群发展、资源集约利用、功能集合构建”的要求，依托能源、铝工业、煤化工、高新技术等主导产业，加快全市7个省级产业集聚区建设，年内完成基础设施投资62.8亿元、工业项目投资280亿元，努力提高产业支撑和人口集聚能力。加快三门峡工业园基础设施建设，抓好创业中心、蓝领公寓等配套设施建设，健全公共服务体系，完善产业集聚区功能，提高园区承载能力和吸引力。引导新上工业项目向园区集中，争取一批高、精、尖的龙头项目落户园区。三是职业教育园区建设。大力实施职业教育攻坚计划，努力争取国家和省级职业教育项目。加快职教园区建设进度，积极筹措资金，协调各方力量，围绕建设全省具有竞争优势的高等职业教育基地，及早做好三门峡中专等学校的搬迁准备，确保园区6月底前开工建设。整合职教资源，重点建设1所特色鲜明、优势明显的省级示范性中等职业学校；加快省级示范性和标准化实训基地建设，改善办学条件，全面促进职业教育提档升级。四是新型农村住宅社区建设。最大限度地聚集、整合各类空间要素，高标准编制村镇规划，按期完成编制任务。坚持规划先行，示范带动，分期分类，压茬推进，上半年完成40个试点社区的建设任务。加强基础设施建设，强化服务体系建设。健全治理机制，有效改善农村面貌。

（四）抓好“六大载体”。一是大交通。加快“三纵四横”大交通网络建设，突出抓好运三高速三门峡黄河大桥及连接线、三淅高速公路、连霍高速改扩建、省道250线卢氏境水毁修复工程等重大交通项目建设，全力争取运（城）十（堰）铁路通过灵宝、卢氏，建设三门峡交通枢纽，形成人流、物流、信息流和资金流集散中心。二是大招商。改进和创新招商形式，主动参加各类大型招商活动，提高招商质量和效益。扎实开展好“千名干部挂职大招商”活动，突出承接产业转移、加强项目合作、搞好技术人才引进“三个重点”，提高招商引资活动的针对性，力争在引进重大合作项目上取得新突破。加强招商引资项目信息库建设，增强引进项目在经济社会发展中的支撑作用。建立健全重大项目协调机制，加强对已签约项目的跟踪落实，提高招商项目的履约率和开工率。三是大商贸。抓住“三纵四横”交通枢纽中心形成的机遇，加大城市商业网点街区建设，加快家乐福、丹尼斯、沃尔玛等大型连锁企业入驻步伐，建设一流大型商贸中心。以郑西高速铁路新三门峡站为依托，加快商业、物流、仓储配送等大型设施建设，培育壮大各类商贸物流市场。四是大旅游。认真实施旅游强市行动纲要，重点打造“一坝（中流砥柱）、一关、一泉、一山和两馆”等重点景区，加强精品景区建设，提升景区品位，提高旅游招徕力。加强与周边城市的合作，打造“黄河金三角旅游精品”线路，实现资源优势互补，增强旅游市场竞争力。拓宽办节思路，及早谋划办好第16届国际黄河旅游节暨投洽会，扩大对外影响力。五是大通关。加快中国河南出入境检验检疫局三门峡办事处、国家铝及铝制品质量监督检验中心建设，加快中国三门峡海关申报，强化大通关体系建设。积极扩大先进技术、装备、关键零部件和国内紧缺物资进口。抓好出口商品基地建设，做好戴卡轮毂出口基地的申报争取工作，加快汽车零部件基地建设，开拓国际高端市场，抢占豫晋陕黄河金三角地区对外开放制高点。六是大合作。进一步拓宽与中国贸促会合作领域，加强与中国黄金集团、中国铝业集团、中国大唐集团等央企的对接合作，加大与法国电力公司、杭州锦江集团、东方希望集团、河南煤化工集团等大型企业合作力度，重点瞄准世界500强以及国内500强、行业50强企业，全面提升合作水平。充分利用国家加快中部崛起的机遇，继续做好豫晋陕黄河金三角综合试验区协调推进工作。巩固政银企战略合作成果，强化与省工农中建四大银行签约资金的跟踪落实，完成年度目标170亿元以上。推进与省地矿局、省有色地矿局的战略合作，提高资源利用效率。加快与省移动公司的战略合作，促进工业化和信息化融合发展。落实与省电力公司的电网发展框架协议，加快推进500千伏三门峡东输变电项目，着力打造坚强智能三门峡电网。

四、统筹城乡协调发展，壮大县域经济实力

（一）继续抓好中心城市建设。继续推进“四城联创”，巩固国家园林城市创建成果，争创国家卫生城市。坚持以紧凑型、复合型理念规划建设城市，加快城市总体规划和新区规划的修编，提高控制性详细规划覆盖面。加强城市规划监察执法力度，严厉打击城市违法占地、违法建设、私搭乱建等行为。加强城市基础设施建设，开通郑西高速铁路新三门峡站到三门峡火车站（汽车站）的迎宾一号公交专线，完善城市交通网络；抓好陕州大道等10条道路的改造和建设，提升道路通行水平。完善城市集中供热二期工程，做好西气东输二线天然气主管道和附属设施建设。稳步推进城中村和旧城改造工程，努力改善城市形象。完善、提升天鹅湖城市湿地公园和涧南公园建设水平，促进人与自然和谐相处。突出抓好城市精细化管理，提高城市基础设施运行效率。

（二）加快推进县域经济发展。大力发展县域经济，突出晋位争先，不断增强县域综合实力。义马、渑池要围绕培育壮大能源、煤化工和铝工业等主导产业，加快城区对接和经济一体化步伐，积极探索城市功能的优势互补，全面推进城区的实质性对接；陕县要以现代制造、农产品加工、高新技术产业为重点，拉长产业链条，加快培育重点骨干企业，做大做强经济支撑；灵宝市要依托现有产业基础，加快新型有色金属冶炼加工和新型果品生产加工两大基地建设，增强辐射带动能力；卢氏县要深入挖掘生态旅游资源，搞好基础设施建设，培育比较优势明显、特色鲜明的支柱产业。积极推进县（市）城区建设，强化产业支撑，引导要素集聚，把县（市）城区建成各具特色的区域性经济、文化中心。坚持走新型工业化、城镇化道路，加快副中心城市和小城镇建设，抓好310国道沿路城镇经济隆起带建设，着力提高小城镇综合承载能力。进一步放宽城镇户籍限制，提高城镇规划水平，解决好符合条件的农村转移人口在城镇的就业和落户问题，全方位提高城乡一体化水平。

（三）切实加强“三农”工作。进一步加大农业支持力度。全面落实各项强农惠农政策，增加对“三农”的投入。稳定实施粮食直补、良种补贴、生猪和奶牛补贴、农机具购置补贴等惠农补贴政策，做好家电下乡、汽车摩托车下乡、以旧换新等工作，刺激农民消费需求，繁荣农村市场。稳定和完善农村基本经营制度，积极探索土地流转模式，逐步建立“政府引导、市场调节、农民自愿、依法有偿”的土地流转机制，力争土地流转面积达到25万亩。大力发展特色农业。稳定粮食生产。提高果品生产的质量和效益，确保苹果出口基地规模达到100万亩，力争苹果、浓缩果汁出口均达到5万吨。巩固提高优质畜产品生产和加工两大基地，重点抓好50万头瘦肉型猪基地建设，推动传统畜牧业向现代畜牧业转变，实现畜牧业产值28亿元。大力发展用材林、经济林、种苗花卉和森林药材等林业产业，拉长产业链条。持续推进烟叶、蔬菜、食用菌、中药材、桑蚕等优势特色产业发展，重点抓好17.3万亩优质烟叶、20万亩特色蔬菜、8000万袋无公害食用菌和渑池万亩玫瑰园基地建设，构建稳定的特色农业产业体系。加快农业“三化”进程。大力扶持壮大农业龙头企业，力争省级农业龙头企业达到18家。积极发展各类农民专业合作社，确保农民专业合作社达到230家，带动农户16万户以上。加大“三品一标”认证力度，严格落实无公害农产品市场准入制度，完善农、畜产品质量检测体系，不断提高农业标准化水平。突出抓好农业信息化服务，着力提升中国苹果网和苹果交易中心的服务水平，增强辐射带动功能，在毗邻城市建立“黄河金三角苹果电子交易网点”。加快农业科技服务体系建设，积极推广先进农业生产技术，加大农机新技术、新机具引进推广力度，农作物良种覆盖率达到95%以上，农业科技进步贡献率达到50%。继续改善农业生产和农民生活条件。统筹安排农业、水利等基础设施建设，加快山口水库复建等项目建设，继续加强农村水电路气建设，启动新一轮农村电网改造。大力开展农田水利基本建设，加强中低产田改造，新增有效灌溉面积3万亩。加快窄口水库等除险加固工程步伐，解决8万人的饮水安全问题。大力发展循环农业，新建农村户用沼气池2万户。扎实开展农业综合开发土地治理，完成治理项目1.5万亩，搬迁扶贫880户、3762人。进一步改善城乡交通条件，建成县乡公路150公里。大力实施“阳光工程”、“雨露计划”和新型农民培训工程，加大培训力度，全年转移农村劳动力30万人次，实现劳务收入21亿元。

五、加快改革创新步伐，优化经济发展环境

（一）扎实推进各项改革。加快政府机构改革步伐，全面完成市、县政府机构改革任务。稳步推进事业单位聘任制和绩效工资改革。巩固国有企业改革成果，全面推进商贸、粮食等流通体制改革。毫不动摇地支持非公有制经济发展，保障非公有制企业公平的市场参与权，营造多种所有制经济公平竞争的市场环境。深化财税改革，主动适应中央积极财政政策和适度宽松货币政策。配合做好政策性银行、国有商业银行、邮政储蓄银行等金融机构改革，重点引进股份制商业银行，支持组建农村商业银行和村镇银行。加快医药卫生体制改革步伐，提高应对突发公共卫生事件能力。全面推进集体林权制度改革，基本完成集体林地明晰产权工作。稳步实施资源性价格和环保收费改革。适应政府职能转变和经济社会发展需要，加快县级供销社改革，积极推进文化、投资、收入分配、教育、科技等领域的改革。

（二）持续增强财政保障。大力培植财源。充分发挥财政职能作用，统筹运用资金、政策、服务等多种手段，不断加大对重点项目、重点企业和产业集聚区的支持力度，着力培植一批骨干财源，增强地方经济发展的保障能力。强化税收征管。细化财税征管责任，完善税源动态监控体系，加强重点税种、重点行业和重点企业的税收管理。进一步加强和规范非税收入管理，优化收入质量和结构，增加可支配财政收入。坚决打击各种偷税、漏税、骗税、逃税和破坏、干扰税收征管秩序的行为，营造良好的税收征管环境。严格支出管理。牢固树立过“紧日子”思想，大力压缩经费开支，不断降低行政成本。进一步优化支出结构，有保有压，加大对“三农”、民生、自主创新、节能环保、技术改造等方面的支持力度，扩大政府采购范围和规模，严格控制一般性支出，把钱花在最需要的地方。

（三）不断优化经济发展环境。坚持诚实守信、有诺必践，创造良好投资环境。综合运用行政、法律、经济和舆论等手段，优化政策环境、法治环境、服务环境、人文环境和信用环境，着力建设优化环境的长效机制，打造“信用三门峡”。加强企业周边和重点项目建设环境治理，整顿和规范市场经济秩序，严厉查处“四乱”及各类违法犯罪行为，营造行为透明、办事高效、服务优良的宽松环境。大力整治教育、医疗乱收费，加大商业贿赂专项治理力度，坚决纠正损害群众利益的不正之风。

六、抓好民生稳定工作，全面促进社会和谐

（一）着力保障和改善民生。坚持把民生工作摆在更加突出的位置，在集中落实好省“十项民生工程”的基础上，继续办好市“十件实事”，下大力气解决好涉及群众利益的现实问题，让更多改革发展成果惠及广大群众。努力扩大社会就业。继续实行积极的就业政策，将就业扶持优惠政策再延长一年。充分发挥政府投资和重大项目带动就业的作用，提高中小企业、劳动密集型产业和服务业吸纳就业的能力。加强创业培训和辅导，通过全民创业带动就业，确保全年城镇新增就业4万人，实现下岗失业人员再就业1.3万人。做好退伍军人转岗安置工作，鼓励引导高校毕业生到基层就业。鼓励和支持劳动者自主创业和自谋职业，用好就业专项资金和失业保险基金，进一步开辟面向就业困难群体的公益性就业岗位，动态消除城镇“零就业家庭”。不断提高社会保障和住房保障水平。继续扩大企业养老、医疗、失业、工伤和生育保险覆盖面，努力解决各类关闭破产企业退休人员和困难企业职工医疗保障问题，实现工伤保险市级统筹。扎实做好新型农村社会养老保险试点工作，完善落实被征地农民补偿机制和社会保障制度。继续提高城乡低保标准，提高企业退休人员基本养老金和部分优抚对象待遇。扩大城镇居民医疗保险范围，参保人员达到30万人以上。进一步加强新型农村合作医疗工作，继续完善覆盖城乡的社会救助体系，加大对低收入群体的帮扶救助力度。加强保障性住房建设，新开工建设经济适用房1810套；加大廉租房租赁补贴发放力度，探索城镇廉租房租售并举办法，有效遏制房价过快增长。加快工矿棚户区改造。建设廉租房2210套、11.05万平方米。

（二）大力发展社会事业。优先发展教育事业，不断改善办学条件，下决心解决好城区大班额问题，促进城乡、区域教育均衡发展；积极整合教育资源，科学调整农村中小学布局，加快农村寄宿制学校建设，继续做好校舍安全工作；促进高等教育、职业教育、成人教育协调发展。加强卫生基础设施建设，提高基层卫生服务水平，完善医院管理评价体系建设，改善群众就医环境。开展“健康快车”活动，免费实施白内障康复手术1000例。完善疾控体系建设，做好甲型H1N1流感、手足口病等重大传染病和地方病的预防控制工作。继续实施文化强市战略，大力发展文化产业。加快豫西文化长廊建设，新建或改建27个乡（镇）综合文化站。加强文化先进县创建工作，力争年底前再创建1个省级文化先进县（市）、10个以上文化先进乡或民间艺术之乡。加强文物保护利用，推进丝绸之路（三门峡段）申遗工作，保护和开发地坑院遗址群，力争仰韶村遗址、庙底沟遗址保护规划尽快通过国家文物局验收。加快广播电影电视基础设施建设，完成 “村村通”广播电视扫尾工程。大力实施农村小康环保行动计划，创建2个环境优美小城镇和20个省级生态文明村。深入开展群众性体育活动，建设96处高标准农民健身场所。做好人口计生工作，综合治理出生人口性别比偏高问题，着力提高出生人口质量。

（三）全力维护社会稳定。深入推进社会管理创新，正确处理各类社会矛盾，健全调节利益关系的社会政策，形成依法有序表达诉求、及时有效解决问题的社会环境，保障群众合法权益。妥善处理土地征用、拆迁安置、环境污染等方面的矛盾和问题，积极预防和处置群体性事件和突发公共事件。加强信访源头治理，认真做好群众工作。深入开展平安创建活动，健全社会治安防控体系，严厉打击各类违法犯罪活动，不断增强人民群众的安全感和满意度。深入开展整顿和规范市场秩序专项行动。加强食品药品安全监管，规范生产经营行为，加快县级食品药品检验所建设，提高食品药品安全保障水平。强化安全生产管理，落实企业主体责任，深入开展隐患排查治理活动，继续深化重点行业和领域的专项整治，抓好小煤矿复工复产工作，加强尾矿库治理监管，确保安全生产形势持续稳定好转。

（四）切实加强精神文明和民主法制建设。深入推进社会主义核心价值体系建设，大力弘扬以爱国主义为核心的民族精神和以改革创新为核心的时代精神，大力弘扬焦裕禄精神、红旗渠精神和愚公移山精神。继续开展以优美环境、优良秩序、优质服务和创建文明城市、文明社区、文明景区为主要内容的“三优三创”竞赛活动，着力提高市民文明素质和城市文明程度。开展清洁家园行动，加强农村精神文明建设。积极开展文明单位结对帮扶和城乡共建精神文明活动，深化文化科技卫生“三下乡”和“四进社区”活动，推动群众性精神文明创建活动蓬勃开展。推进社会文化环境净化工程，加强以互联网、音像制品为重点的文化市场管理，深化未成年人思想道德建设。加强社区建设，完善村民自治。加强法制宣传教育，积极开展法律援助。

同时，继续加强国防教育和国防后备力量建设，深入开展双拥共建活动，不断提高预备役部队、民兵和人民防空建设质量，营造和谐的军政、军民关系。支持工会、共青团、妇联等人民团体发挥桥梁纽带作用，密切联系群众，积极参与社会事务管理和公共服务。切实做好民族、宗教、外侨、对台、史志、气象、防震减灾等工作。全面完成江油援建任务。

七、扎实推进“两转两提”，加强政府自身建设

（一）继续转变政府职能。围绕服务型政府建设，进一步减少和规范行政审批事项，切实简政放权，减少政府对微观经济的干预，激发和释放市场主体的活力。加强和规范行政服务中心建设，完善联审联批、绿色通道、全程代办、特事特办等便民措施。建设行政效能电子监察系统，实现对行政审批、行政征收、行政处罚等施政情况的实时监控、预警纠错和绩效考评，不断提高政府部门及其工作人员的服务意识和服务水平。加强应急队伍建设，完善应急管理体系，提升应急保障能力。

（二）全面推进依法行政。严格执行人大及其常委会的决议、决定，自觉接受人大及其常委会的法律监督和工作监督。自觉接受人民政协的民主监督。广泛听取各民主党派、工商联和无党派人士的意见和建议。坚持依法行政，理顺执法体制，强化执法责任，推进文明执法。加强行政复议工作。支持监察、审计等部门依法独立履行职责。依照有关法律的规定，接受司法机关的监督。重视发挥新闻舆论和社会公众的监督作用。加强政府信息公开，充分保障人民群众的知情权、参与权、表达权和监督权。完善行政问责制度，坚决防止行政不作为和乱作为。

（三）切实加强廉政建设。严格落实党风廉政建设责任制，围绕重点领域、重点工程和重点环节，完善惩治和预防腐败体系，强化权力运行制约和监督，依纪依法严肃查处权钱交易、失职渎职、侵害群众利益等案件。坚持厉行节约，制止铺张浪费，做到令行禁止、违者必究。各级领导干部和政府工作人员都要做到廉洁自律、公道正派，做遵纪守法的模范、廉洁奉公的表率。

（四）积极转变工作作风。继续坚持解放思想，扎实推进“两转两提”，深入开展“做人民满意的公务员”活动和“机关作风效能评议”活动，切实提高政府公信力、贯彻力和执行力。坚持科学决策，增强前瞻性、系统性，力争政府工作少走弯路、少走错路。坚持深入实际、深入基层、深入群众，做到问政于民、问需于民、问计于民。严格控制各类检查、评比，从简、务实地举办节庆活动，腾出更多的精力谋大事、谋发展。发扬求真务实和艰苦奋斗精神，努力做出经得起实践、人民和历史检验的实绩。

各位代表，面对改革发展的新形势，面对全市人民的新期待，我们深感责任重大，使命光荣。让我们在省委、省政府和市委的正确领导下，团结拼搏，乘势而上，为促进经济社会平稳较快发展、跻身中原崛起第一梯队而努力奋斗！
